# Identification of four potential predicting miRNA biomarkers for multiple myeloma from published datasets

**DOI:** 10.7717/peerj.2831

**Published:** 2017-01-31

**Authors:** Tian Xiang, Ai-Xin Hu, Peng Sun, Gao Liu, Gang Liu, Yan Xiao

**Affiliations:** 1 Department of Clinical Laboratory Center, Central Hospital of Enshi Autonomous Prefecture, Enshi Clinical College of Wuhan University, Enshi, Hubei, China; 2 The Department of Orthopedic Surgery, People’s Hospital of Three Gorges University, YiChang, Hubei, China; 3 Department of Orthopedics, Huai’an First People’s Hospital, Nanjing Medical University, Huai’an, China; 4 Department of Gastrointestinal Surgery, Central Hospital of Enshi Autonomous Prefecture, Enshi Clinical College of Wuhan University, Enshi, Hubei, China; 5 Department of Hematology, The Affiliated Huai’an Hospital of Xuzhou Medical College and The Second People’s Hospital of Huai’an, Huai’an, China

**Keywords:** Multiple myeloma, Mirna, Signature, Enrichment analysis

## Abstract

**Background:**

Multiple myeloma is a cancer which has a high occurrence rate and causes great injury to people worldwide. In recent years, many studies reported the effects of miRNA on the appearance of multiple myeloma. However, due to the differences of samples and sequencing platforms, a large number of inconsistent results have been generated among these studies, which limited the cure of multiple myeloma at the miRNA level.

**Methods:**

We performed meta-analyses to identify the key miRNA biomarkers which could be applied on the treatment of multiple myeloma. The key miRNAs were determined by overlap comparisons of seven datasets in multiple myeloma. Then, the target genes for key miRNAs were predicted by the software TargetScan. Additionally, functional enrichments and binding TFs were investigated by DAVID database and Tfacts database, respectively.

**Results:**

Firstly, comparing the normal tissues, 13 miRNAs were differently expressed miRNAs (DEMs) for at least three datasets. They were considered as key miRNAs, with 12 up-regulated (hsa-miR-106b, hsa-miR-125b, hsa-miR-130b, hsa-miR-138, hsa-miR-15b, hsa-miR-181a, hsa-miR-183, hsa-miR-191, hsa-miR-19a, hsa-miR-20a, hsa-miR-221 and hsa-miR-25) and one down-regulated (hsa-miR-223). Secondly, functional enrichment analyses indicated that target genes of the upregulated miRNAs were mainly transcript factors and enriched in transcription regulation. Besides, these genes were enriched in multiple pathways: the cancer signal pathway, insulin signal metabolic pathway, cell binding molecules, melanin generation, long-term regression and P53 signaling pathway. However, no significant enrichment was found for target genes of the down-regulated genes. Due to the distinct regulation function, four miRNAs (hsa-miR-19a has-miR-221 has-miR25 and has-miR223) were ascertained as the potential prognostic and diagnostic markers in MM. Thirdly, transcript factors analysis unveiled that there were 148 TFs and 60 TFs which bind target genes of the up-regulated miRNAs and target genes of the down-regulated miRNAs, respectively. They respectively generated 652 and 139 reactions of TFs and target genes. Additionally, 50 (31.6%) TFs were shared, while higher specificity was found in TFs of target genes for the upregulated miRNAs.

**Discussions:**

Together, our findings provided the key miRNAs which affected occurrence of multiple myeloma and regulation function of these miRNAs. It is valuable for the prognosis and diagnosis of multiple myeloma.

## Introduction

Multiple myeloma (MM), a hematological malignancy, is characterized by aberrant clonal proliferation and expansion of malignant bone marrow plasma cells (Gulla et al., 2016; Noll et al., 2014). MM is also one of the most common hematologic malignancies, only second to non-Hodgkin’s lymphoma (Siegel, Naishadham & Jemal, 2012), and accounts for nearly 13% of all hematologic cancers (Ghobrial et al., 2016). Although great advances have been made in the understanding of the MM pathogenesis and the development of potential effective therapies, it still remains incurable (Zhao et al., 2014). Therefore, there is urgent need for discovering and developing.

Novel and more promising therapeutic targets or treatments in MM. miRNAs, a class of endogenous short, non-coding single-stranded regulatory RNA molecules with 18–25 nucleotides in length, can regulate target gene expression at the post-transcriptional level via binding to 3′ untranslated region (3′ UTR) of specific messenger RNAs (Chen et al., 2015; Li et al., 2012). Recent studies have confirmed the abnormal miRNA expression in various cancer types including MM, and miRNAs were found to be served as tumor suppressors to regulate cell proliferation, migration, cell cycle, differentiation and so on (Ma et al., 2014; Zhao et al., 2015). For example, MiR-15a, miR-16-1 and miR-17-92 expression are proved to be involved in the poor prognosis of MM (Gao et al., 2012). miR-186 is found to inhibit cell proliferation by repressing Jagged1in MM (Liu et al., 2016). While miR-320a has been reported to regulate cell proliferation and apoptosis in MM through targeting pre-B-cell leukemia transcription factor 3 (Lu et al., 2016).

In spite of the rapid development in miRNA profiling studies in recent years, the variations in different studies and the small sample size leaded to the poor repeatability of the results. In this study, we explored the miRNA expression and identified the differentially expressed miRNAs (DEMs) in MM. We hope to find the miRNAs associated with MM pathogenesis and predict their target genes, in addition, we analyzed the functions of the target genes regulated by the DEMs in MM, which may contribute to the prevention and treatment of MM.

## Material and Methods

### Identification of MM-related datasets and screening of DEMs

The articles about MM miRNA expression were retrieved on the Google Academic website using search terms “multiple myeloma” and “miRNA.” We carefully evaluated these articles; only the studies that provided detailed information on the sequencing samples of MM miRNAs and control samples were selected for subsequent analysis. The detailed information of the DEMs in MM was extracted compared with the control samples. We also calculated the times that the up-regulated and down-regulated miRNAs appeared in the independent datasets respectively, and the reserved condition was set as appearance in at least three separate datasets according to the distribution of DEMs in datasets.

### Target gene prediction of DEMs in MM

The sequence information of the DEMs was identified in miRBase database (Dennis et al., 2003; Lewis, Burge & Bartel, 2005) and these miRNA sequences were reorganized into fasta files. The target genes corresponding to the DEMs were predicted using TargetScan software (Lewis, Burge & Bartel, 2005), and the default parameters were adopted.

### Functional enrichment analysis of the target genes of DEMs in MM

The predicted target genes of the DEMs were mapped to DAVID database (Dennis et al., 2003), and the Gene Ontology (GO) annotation and pathway results of these genes were analyzed. The top 10 GO terms in biological processes were extracted as GO annotation results, and the corrected *P* value (Benjamini) was required to be no more than 0.05.

### Analysis of the transcription factors regulating the target genes of the up or down-regulated miRNAs

The target genes of the up or down-regulated miRNAs in MM were then submitted to Tfacts database (Essaghir et al., 2010), a site at: http://www.tfacts.org/. *p*-value, *q*-value, *E*-value and FDR were all applied for prediction of transcription factors, and only the transcription factor with these four indexes all less than 0.05 was considered to be reliable. The transcription factor numbers of the target genes regulated by the up or down-regulated miRNAs were calculated respectively, furthermore the common and specific transcription factors between these target genes were compared.

## Results

**Figure 1:**
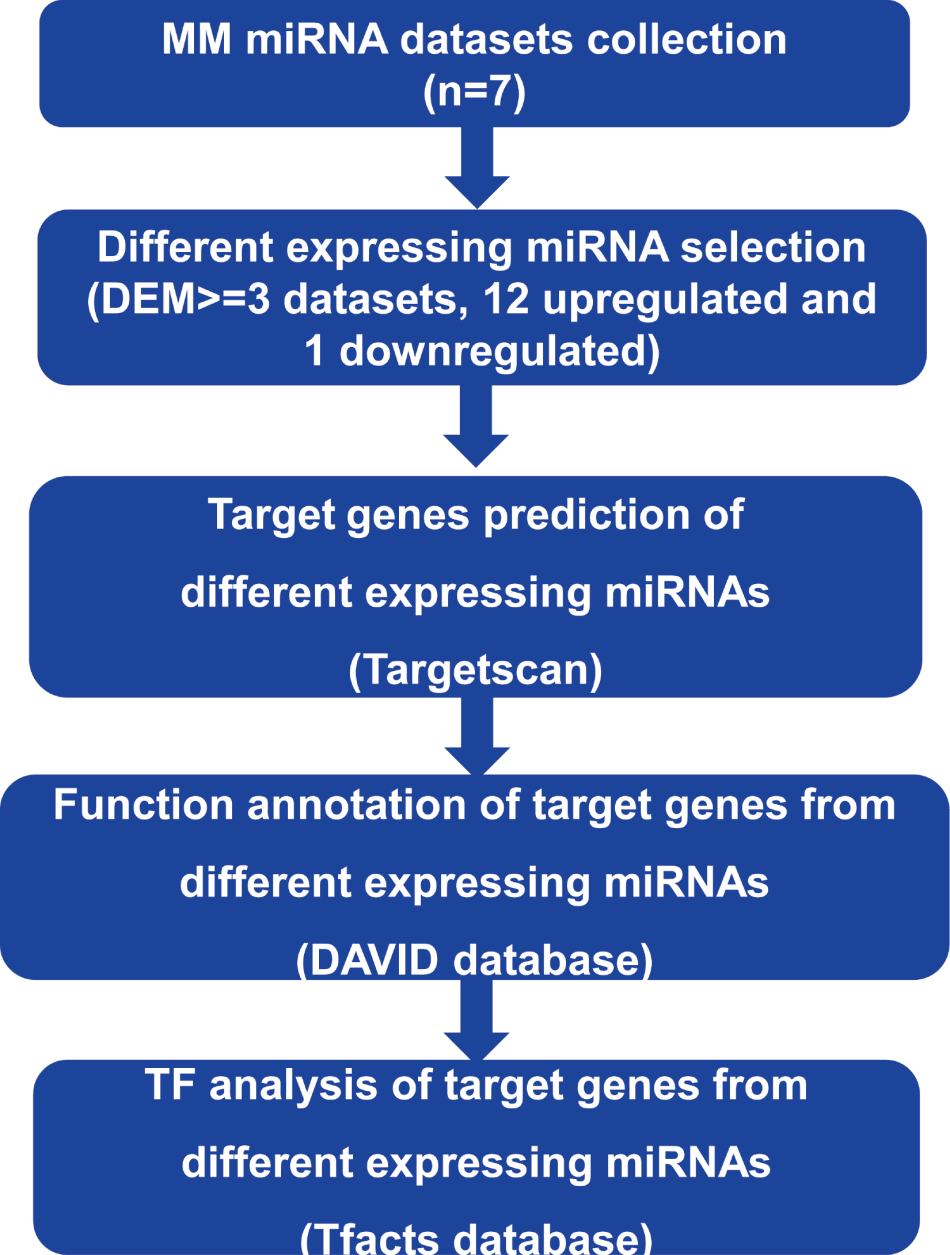
The analysis process in this study. The key DEMs were determined by comparing the distribution of different expressed miRNAs among multiple datasets. Then, target genes of DEMs were predicted by Targetscan. Finally, function enrichment and binding TFs were analyzed by DAVID and Tfacts databases, respectively.

In order to analyze the key miRNAs and their target genes in MM and explore its pathogenesis, we identified the critical up or down-regulated miRNAs in the existing MM dataset. We also predicted the target genes and performed functional annotation and transcription factor analysis of the target genes, as shown in Fig. 1.

### Screening of DEMs

**Figure 2:**
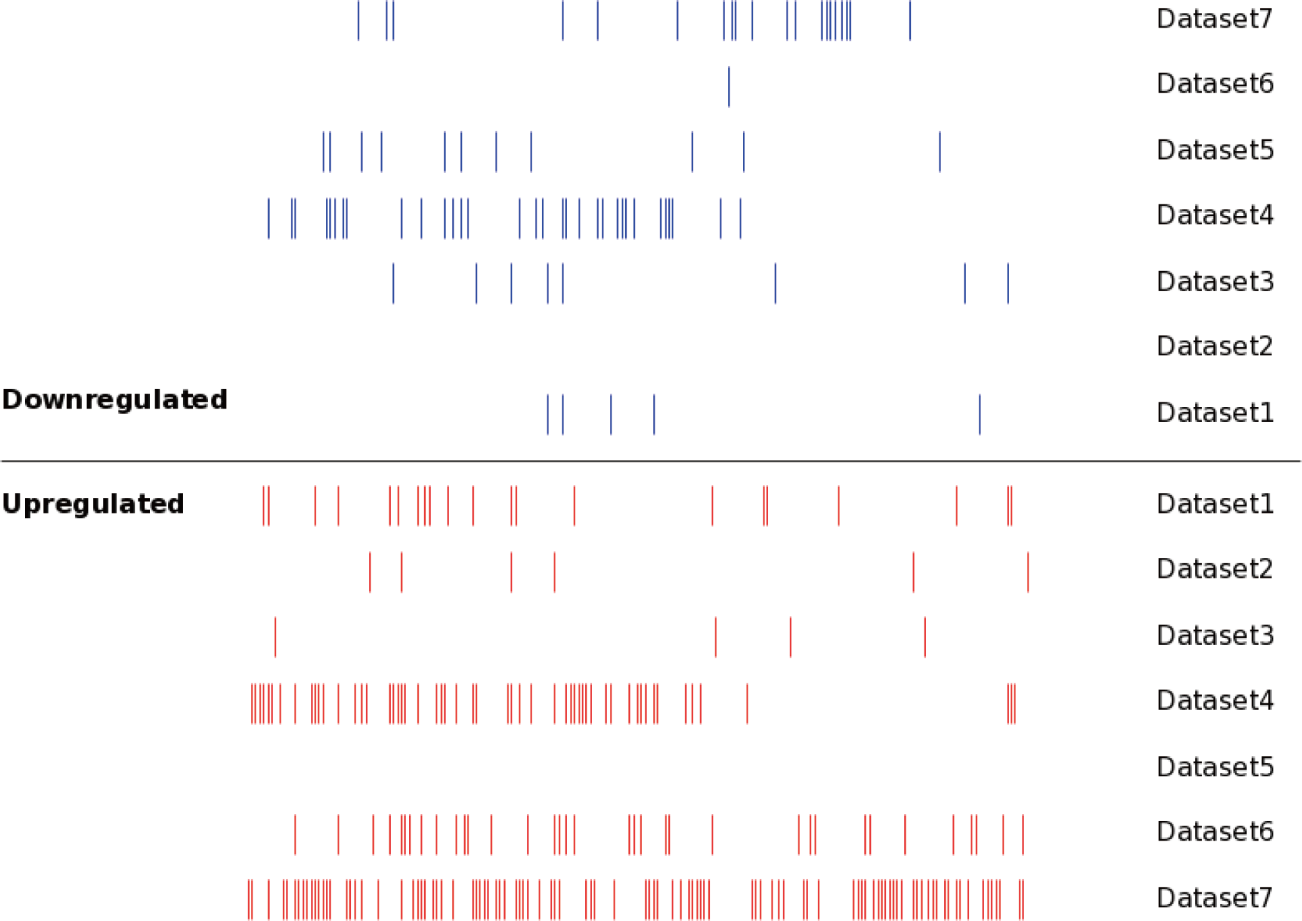
The distribution of the DEMs in seven datasets. Each line represented an independent miRNA in respective dataset. The red line presented an upregulated miRNA, while the blue line presented a downregulated miRNA. The miRNAs with the same position of *x* axis were the identical miRNAs. The DEMs showed significant differences between different datasets.

Seven independent miRNA expression datasets were identified in the existing MM miRNA expression profiles, these datasets provided detailed miRNA expression results of MM tissues. For the convenience of study, we numbered the 7 MM miRNA datasets, and the specific information was as follows: dataset 1 (Unno et al., 2009); dataset 2 (Huang et al., 2012); dataset 3 (Huang et al., 2012); dataset 4 (Pichiorri et al., 2008); dataset 5 (Gutierrez et al., 2010); dataset 6 (Zhou et al., 2010); dataset 7 (Zhou et al., 2010). The main characteristics of these seven datasets were shown in Table 1, which included sample type, sample size, chip type and probe number of the chip. The identified DEMs in the seven datasets were verified by a qPCR experiment respectively, indicating the results of DEMs obtained in various studies were reliable. We still analyzed the distribution of the DEMs in seven datasets using SVG graphic, and found the DEMs showed significant differences between datasets (Fig. 2).

**Table 1:** The basic characteristics of MM datasets.

Dataset	Samples	Assay type	miRNA probes	Validated
1	138 positive plasma cells (MM), 138 positive plasma cells [healthy donors]	miRCURY™ LNA	757	qRT-PCR
2	The plasma of 12 multiple myeloma patients and 8 healthy controls	TaqMan low-density	667	qRT-PCR
3	The plasma of 40 myeloma patients and 20 healthy controls	Agilent	–	qRT-PCR
4	MM-derived cell lines (*n* = 49) and CD138 bone marrow PCs from subjects with MM (*n* = 16), normal donors (*n* = 6)	Ohio State University custom	−1,000	qRT-PCR
5	60 patients, 5 healthy controls of bone marrow (BM) samples	TaqMan	368	qRT-PCR
6	CD138+ plasma cell samples of 52 patients newly diagnosed with MM were compared to those in samples from two healthy donors	Affymetrix human genome U133Plus 2.0	–	qRT-PCR
7	CD138+ cells from 33 patients, ten controls.	Agilent	655	qRT-PCR

**Figure 3:**
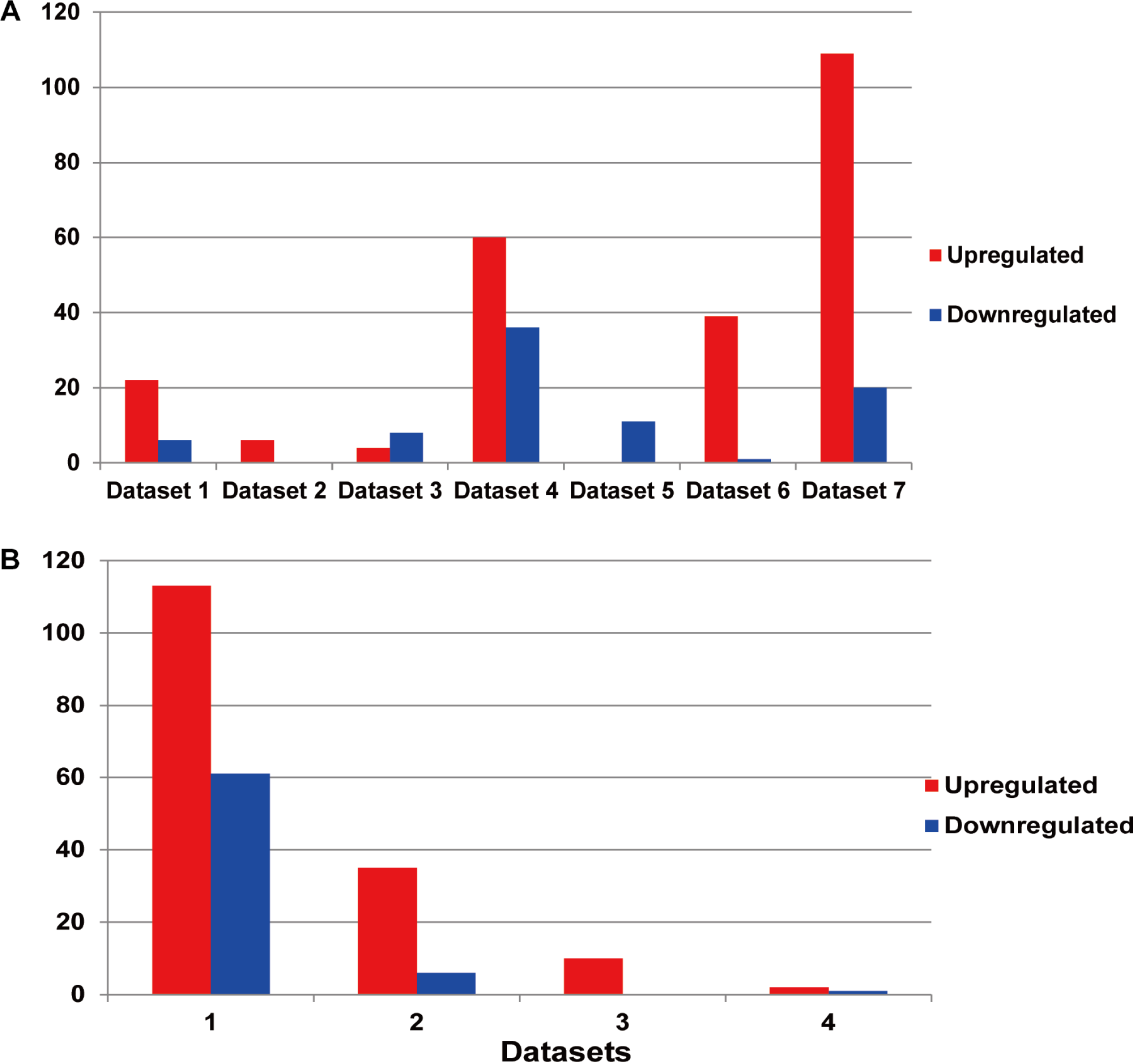
(A) Statistics of the DEMs in seven independent datasets. Red represented the number of up-regulated miRNAs compared with the normal samples, while blue showed the number of don-regulated miRNAs. (B) Statistics of the up or down-regulated miRNAs in seven independent datasets. The *x* axis represented the number of datasets, while the *y* axis represented the number of different expressed miRNAs. Most DEMs were specific (i.e., only appeared in one study).

Among the existing seven MM miRNA expression datasets, the total numbers of the DEMs varied (Fig. 3A). There were 4 datasets possessing more than 30 DEMs, while the other 3 had less than 20, and they were 6 (dataset 2), 12 (dataset 3) and 11 (dataset 5) respectively. Dataset 7 had the most of up-regulated miRNAs (109), followed by dataset 4 (60), dataset 5 had the least (0). Among the down-regulated miRNAs, dataset 4 ranked the first (36), then the dataset 7 (20). No down-regulated miRNAs in dataset 2.

### Identification of the DEMs

The DEM information extracted from the corresponding literature was shown in Fig. 3B. A total of 160 non-redundant up-regulated and 68 down-regulated miRNAs were identified in the seven independent datasets. And these DEMs were supported by different number of datasets. As for the up-regulated miRNAs, the total number of dataset specific miRNAs was 113, accounting for 70.6% of the total non-redundant up-regulated miRNAs. While for the down-regulated miRNAs, there were 61 dataset specific miRNAs accounting for 89.7% of the total. Whether the up-regulated or down-regulated miRNAs, the numbers of the dataset specific miRNAs were all more than 70% of the non-redundant miRNAs, which suggested the sequencing results of the existing MM expression profile were of great difference, at the same time confirmed the necessity that we analyzed the DEMs in MM.

The DEM supported by at least three datasets was considered as a reliable DEM. Based on this standard, 12 up-regulated and one down-regulated miRNAs were finally identified in MM DEMs, and these 13 miRNAs were believed to be highly reliable.

Furthermore, we obtained the detailed location of miRNAs in miRBase database (shown in Table 2). There were 13 DEMs, most of them were located at chromosome 3 and 7 (3 respectively), each accounted for 23.1% of the DEMs, followed by chromosome 13 and X (2 respectively). There was one DEM located at chromosome 9, 21 and 22, respectively.

**Table 2:** MM meta- signature miRNAs.

miRNA	Chromosome	Beg	End	Strand	Sequence
Upregulated					
hsa-miR-106b	chr7	100094043	100094063	−	UAAAGUGCUGACAGUGCAGAU
hsa-miR-125b	chr21	16590253	16590274	+	UCCCUGAGACCCUAACUUGUGA
hsa-miR-130b	chr22	21653316	21653336	+	ACUCUUUCCCUGUUGCACUAC
hsa-miR-138	chr3	44114234	44114256	+	AGCUGGUGUUGUGAAUCAGGCCG
hsa-miR-15b	chr3	160404607	160404628	+	UAGCAGCACAUCAUGGUUUACA
hsa-miR-181a	chr9	124692480	124692502	+	AACAUUCAACGCUGUCGGUGAGU
hsa-miR-183	chr7	129774967	129774988	−	UAUGGCACUGGUAGAAUUCACU
hsa-miR-191	chr3	49020672	49020694	−	CAACGGAAUCCCAAAAGCAGCUG
hsa-miR-19a	chr13	91350904	91350925	+	AGUUUUGCAUAGUUGCACUACA
hsa-miR-20a	chr13	91351072	91351094	+	UAAAGUGCUUAUAGUGCAGGUAG
hsa-miR-221	chrX	45746221	45746242	−	ACCUGGCAUACAAUGUAGAUUU
hsa-miR-25	chr7	100093610	100093630	−	AGGCGGAGACUUGGGCAAUUG
Downregulated					
hsa-miR-223	chrX	66018895	66018916	+	CGUGUAUUUGACAAGCUGAGUU

### Target gene prediction of the DEMs

The target genes of 13 DEMs were predicted using TargetScan software. Although default parameters were applied, significant difference appeared in the target gene prediction results. Accordingly the maximum number of the target genes was limited at 500 so as to accurately evaluate the target genes regulated by the DEMs. The target gene number of four DEMs including hsa-miR-19a, has-miR-221, has-miR25 and has-miR223 reached to 500, the other nine DEMs achieved 100. The number of target genes reflected the various conservatism of DEMs, and the target gene number was proportional to the conservatism. We inferred the four miRNAs (hsa-miR-19a, has-miR-221, has-miR25 and has-miR223) were highly conservative in their sequences, while the other nine showed similar or the same level of conservatism.

### Functional analysis of the target genes

**Figure 4:**
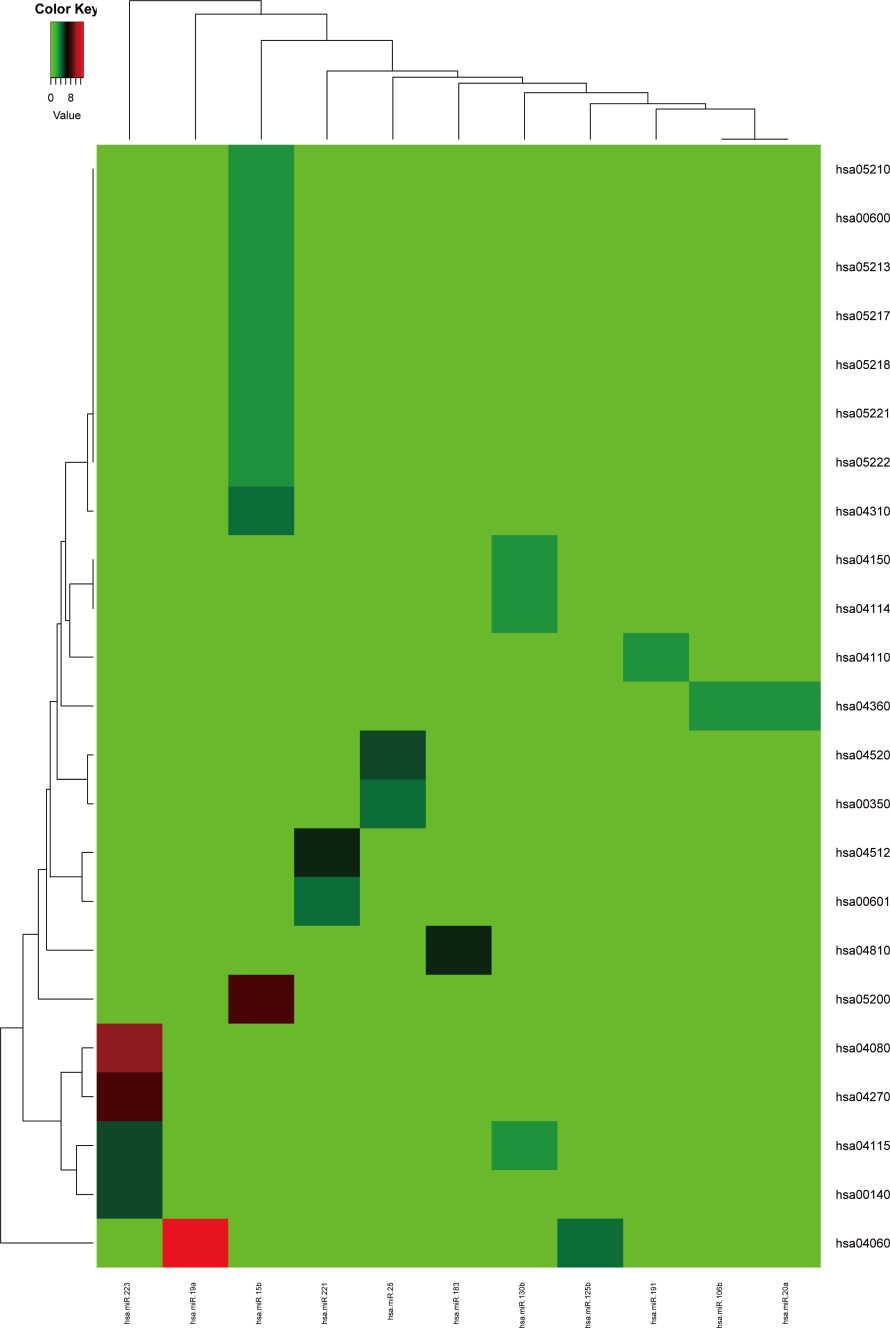
The metabolic pathways enriched by the target genes of meta-signature miRNAs. Among the 13 DEMs, the target genes of 11 DEMs showed corresponding pathways enriched. These pathways were different with each other, the pathways of the target genes of hsa-miR-15b had the most of GO terms and highest enrichment level. The anamorphism from green to red represented the target gene number of the corresponding pathway terms, the deep the color, the high the proportion.

Firstly, the metabolic pathways enriched by the target genes of miRNA were extracted, the results were displayed in Fig. 4. A total of 11 target genes of 12 DEMs showed functional pathway enrichment results, and the enrichment level of metabolic pathways varied. Moreover, the pathway enrichment of target genes of hsa-miR-15b was the most extensive and showed the highest enrichment degree.

The target genes of the up or down-regulated miRNAs were subjected to do functional annotation analysis by DAVID. And the top 10 significantly differential biology processes corresponding to the target genes regulated by the dysregulated miRNAs were obtained respectively. Furthermore, the pathways with corrected P value less than 0.05 were screened, the result was listed in Table 3.

**Table 3:** The GO annotation results of the target genes regulated by the up-regulated miRNAs in MM samples.

Term	Count	*P*-value	Fold enrichment	Benjamini	FDR	Fisher exact
Regulation of transcription	375	3.60E−16	1.5	1.20E−12	6.10E−13	2.00E−16
Regulation of transcription, DNA-dependent	270	3.90E−14	1.5	7.20E−11	7.10E−11	2.40E−14
Regulation of RNA metabolic process	274	6.00E−14	1.5	7.50E−11	1.10E−10	3.70E−14
Transcription	306	1.60E−13	1.5	1.50E−10	3.00E−10	1.00E−13
Pattern specification process	49	3.30E−05	1.8	2.40E−02	6.10E−02	1.60E−05
Regionalization	39	4.50E−05	2	2.80E−02	8.30E−02	2.00E−05
Anterior/posterior pattern formation	30	9.50E−05	2.2	4.90E−02	1.80E−01	3.80E−05
Regulation of cell proliferation	111	1.10E−04	1.4	5.00E−02	2.00E−01	7.40E−05

From Table 3, we could see the up-regulated miRNAs in MM were mainly enriched in transcription regulation and RNA metabolism regulation, indicating most of their target genes were transcription factors, miRNA may strengthen the regulation of transcription factor so as to increase the regulation of gene expression by transcription factors. There was no significant enrichment in the target genes regulated by the down-regulated miRNAs.

**Figure 5:**
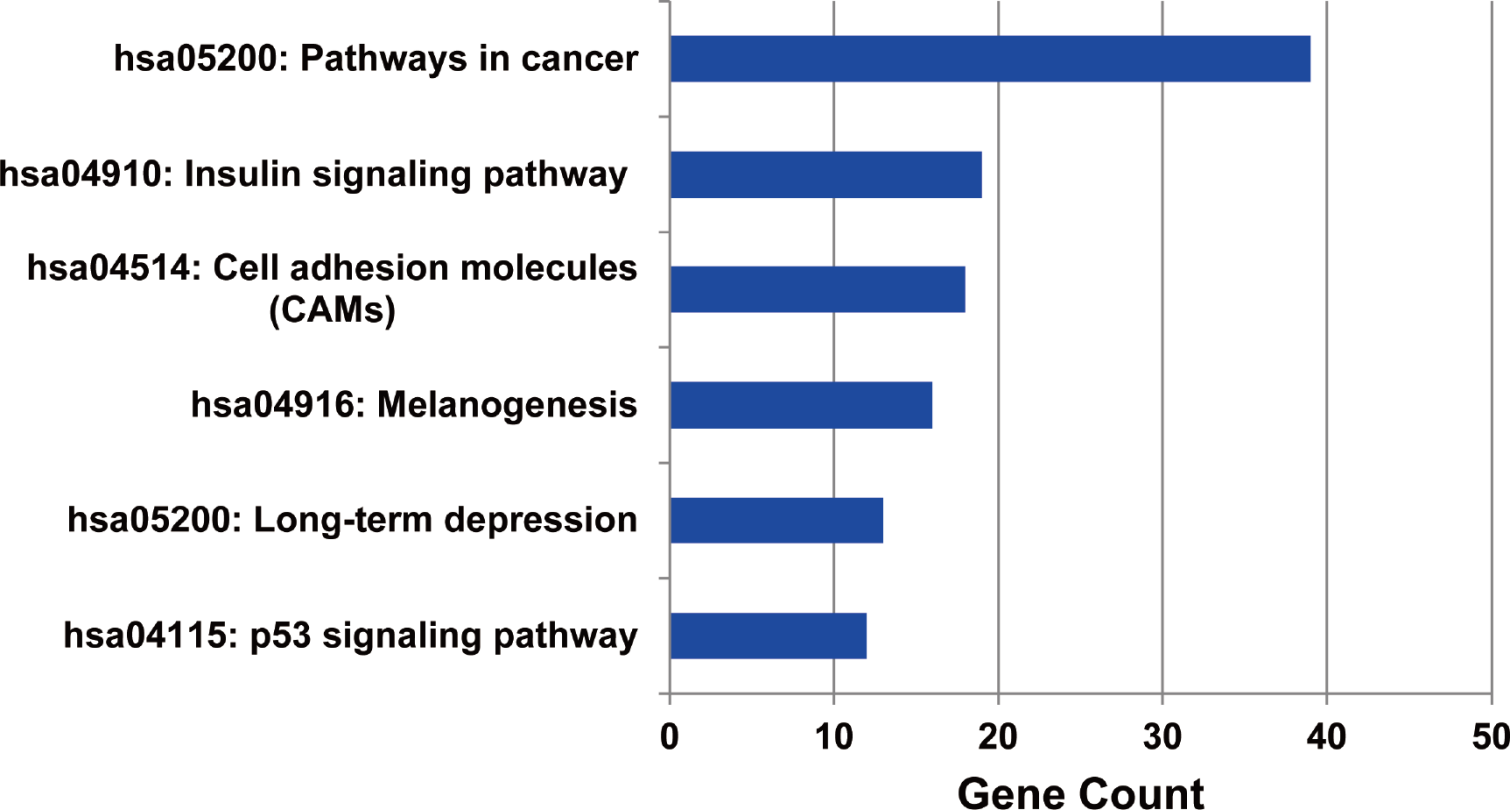
The metabolic pathways enriched by the target genes of up-regulated miRNAs in MM. The target genes of up-regulated miRNAs were enriched in six metabolic pathways, and highly enriched in cancer pathway.

The metabolic pathways enriched by the target genes of miRNA were further extracted. We found the target genes of the up-regulated miRNAs were mainly enriched in six metabolic pathways including cancer signal pathway, insulin signal metabolic pathway, cell binding molecules, melanin generation, long-term regression and P53 signaling pathway (as shown in Fig. 5), and the number of genes enriched in cancer signaling pathway was the largest, suggesting that the up-regulated miRNAs affected pathogenesis process of MM through a variety of ways and participating in various metabolic pathways after regulating the expression of target genes. We also analyzed the metabolic pathways enriched by the target genes of down-regulated miRNAs; however, there was no significant enrichment.

### Transcription factor analysis of the target genes regulated by the DEMs

**Figure 6:**
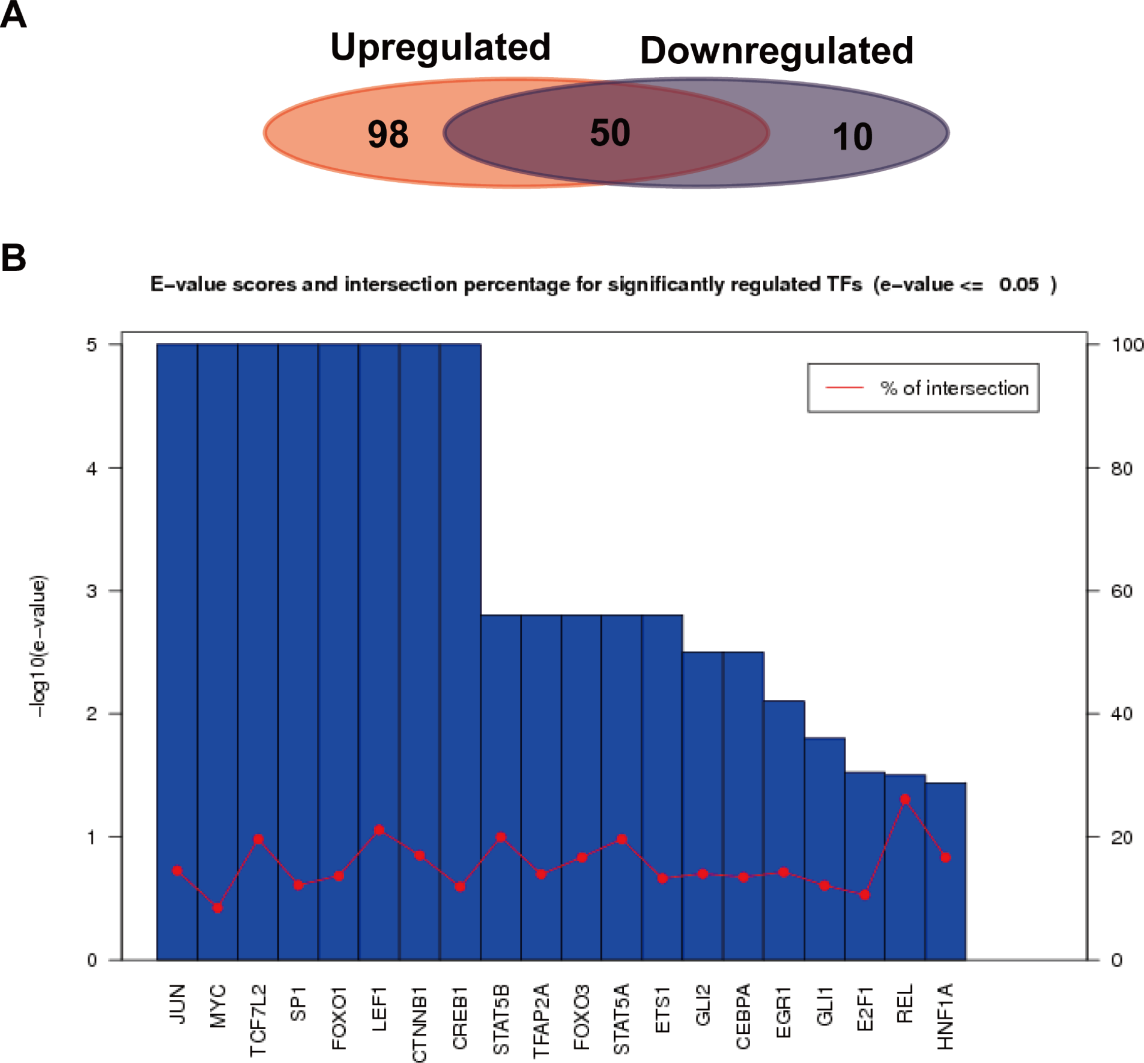
Transcription factor analysis of the target genes of the up or down-regulated miRNAs. (A) Comparison of the common and specific transcription factors for targets genes of the up or down-regulated miRNAs. The red background represents the transcription factor number of the target genes of the up-regulated miRNAs. The blue background shows that of the down-regulated miRNAs. (B) Statistics of *E*-values and cross ratios of the important regulating transcription factors. The highest intersection for predicted TFs was 30% at REL between target genes of upregulated miRNAs and downregulated miRNA; this suggests the high confidence of TF prediction.

We further analyzed the transcription factors corresponding to the target genes of the up or down-regulated miRNAs, and compared the similarities and differences of transcription factors corresponding to the two kinds of target genes. As shown in Fig. 6A, a total of 652 interactions formed between 148 transcription factors and target genes in the up-regulated miRNAs, while 139 interactions formed in the down-regulated miRNAs. Their similarities and differences were compared by a Wayne figure; 50 of 158 transcription factors were shared by the two types of target genes, accounted for 31.6% of the total transcription factors. The transcription factors of target genes in the up-regulated miRNAs displayed higher specificity, which was consistent with GO annotation result, as the regulated transcription was significantly enriched in the target genes of the up-regulated miRNAs. Figure 6B showed the *E*-value and cross ratio of the main transcription factors, and their main *E*-values were all no more than 0.05 and the highest cross ratio was less than 30%. All the results above proved that the transcription factors identified were reliable.

## Discussions

It is all known that miRNAs are related to various important biological processes like cell differentiation and growth (Bartel, 2004). The miRNA expression profiles have been explored in many hematological malignancies, including chronic lymphocytic leukemia (Calin et al., 2004) and acute myeloid leukemia (Jongen-Lavrencic et al., 2008); nevertheless, the available information for MM is limited (Pichiorri et al., 2008). Although a number of studies have applied high-throughput technologies to explore miRNA expression levels between normal and MM tissues and some specific DEMs have been identified, the result reproducibility is not satisfactory. We took a method to investigate the miRNA expression profile and identify the dysregulated miRNAs in MM based on the present published miRNA data of MM, which could combine the results of various individual studies so as to improve the statistical level and avoid the limitations of other analysis approaches.

In the study, a total of 13 DEMs including 12 up-regulated and one down-regulated miRNAs were identified. Among these 13 DEMs, there were four miRNAs (hsa-miR-19a, has-miR-221, has-miR25 and has-miR223) highly conservative in sequence and their target gene numbers were the most. Furthermore, the four miRNAs had already been reported to be functionally important in several cancer types including MM in the previous studies. For example, Hao et al. (2015) found that low expression of miR-19a in serum could be considered as a new poor prognostic marker in MM. They revealed that the serum level of miR-19a may be one of indicators to distinguish myeloma patients from health people, and their research results showed the miR-19a expression varied with myeloma progression, indicating miR-19a as a diagnostic tool for myeloma identification. Similarly, miR-19a was also proved to be a diagnostic tool for MM identification. The miR-19a expression was discovered to display a negative impact on patients’ survival and decreased miR-19a expression contribute to a shorten PFS, which may prove the prognostic role of miR-19a in patients with MM. Up-regulation of miR-221 was found to induce malignant human osteosarcoma, suggesting that miR-221 may be used as a target for osteosarcoma therapy (Yang et al., 2015). They reported that miR-221 up-regulation could promote osteosarcoma cell survival though a PI3K/Akt pathway and reduce its apoptosis (Zhao et al., 2013). Moreover, accumulating evidence had indicated the function of miR-221 during various human malignancy tumorigenesis such as glioblastoma, hepatocellular carcinoma, gastric cancer and so on (Guo et al., 2010; Liu et al., 2012; Quintavalle et al., 2012). Enforced expression of miR-221/222 inhibitors was also reported to trigger *in vitro* anti-proliferative effects and up-regulation of canonic miR-221/222 targets in MM cells highly expressingmiR-221/222. Also, significant anti-tumor activity was achieved in xenografted mice by the treatment with miR-221/222 inhibitors, together with up-regulation of canonic protein targets in tumors retrieved from animals (Di Martino et al., 2013). In addition, Di Martino et al. (2014) demonstrated that silencing of miR-221/222 with an antisense oligonucleotide (ASO) inhibits proliferation of MM cells *in vitro* and significantly slows the tumor growth in xenografted non-obese diabetic/severe combined immunodeficient (NOD.SCID) mice. miR-25, a member of miR-106b-25 cluster, was also known to be dysregulated in several cancer types (Li et al., 2013). Increased expression of miR-25 was reported to be associated with liver, prostate and stomach cancers (Kim et al., 2009; Li et al., 2009; Poliseno et al., 2010). In these cancers, miR-25 acted as an oncogene and exhibited proliferative or anti-apoptotic effects (Li et al., 2009; Razumilava et al., 2012). Finally, miR-223 was discovered to regulate the myeloid cell recruitment and neutrophil-driven lethal inflammation (Dorhoi et al., 2013). Liu et al. (2012) reported that miR-223 had the ability to suppress the activation of NF-*κ*b and nuclear translocation in MDM.

The functions of the target genes for the MM related DEMs were also studied. The results showed the target genes of up-regulated miRNAs were significantly enriched in the transcriptional regulation processes, which indicated miRNA could further regulate the expression of some genes through the regulation of transcription factors, and the regulation of MM incidence was a comprehensive feedback process. The target genes regulated by the up-regulated miRNAs were enriched in a variety of metabolic pathways; the biological significance of cancer signaling pathways and P53 signaling pathways during the MM pathogenesis remained to be further studied.

To conclude, we systematically analyzed the miRNA expression profile datasets in MM. Twelve up-regulated miRNAs and one down-regulated miRNA were identified. Four DEMs (hsa-miR-19a, has-miR-221, has-miR25 and has-miR223) may be considered as potential prognostic and diagnostic markers in MM.

##  Supplemental Information

10.7717/peerj.2831/supp-1 Data S1 Raw data of miRNA targets

10.7717/peerj.2831/supp-2 Table S1 DEM distribution for the seven Multiple myeloma datasets

10.7717/peerj.2831/supp-3 Data S2 Raw data of TF list of miRNA targets

## Additional Information and Declarations

### Data Availability

The following information was supplied regarding data availability: The raw data has been supplied as a Supplementary File.
